# Comparative pharmacokinetics of periplocin and its four key metabolites in rats after oral administration of periplocin and cardiac glycoside extract from *Cortex Periplocae*

**DOI:** 10.3389/fphar.2026.1844009

**Published:** 2026-05-14

**Authors:** Peng Zhao, Fanjiao Zuo, Caixia Li, Yingjing Zhao, Yanjin Li, Haoran Wu, Yameng Zhu, Jun He

**Affiliations:** State Key Laboratory of Chinese Medicine Modernization, Tianjin University of Traditional Chinese Medicine, Tianjin, China

**Keywords:** comparative pharmacokinetics, *Cortex Periplocae*, metabolites, periplocin, UPLC-TQ-S-MS/MS

## Abstract

**Introduction:**

*Cortex Periplocae* has been demonstrated to possess a variety of pharmacological effects, particularly in the treatment of chronic heart failure, attributed largely to its cardiac glycosides particularly periplocin.

**Methods:**

In order to gain a deeper understanding of the pharmacokinetic behaviors of periplocin and its metabolites, and to elucidate the influence of the cardiac glycoside extract matrix on their dynamic process *in vivo*, an ultra-performance liquid chromatography-triple quadrupole mass spectrometry (UPLC-TQ-S-MS/MS) method was developed and successfully applied to a comparative pharmacokinetic study in rats following oral administration of either pure periplocin (50 mg/kg) or cardiac glycoside extract from *Cortex Periplocae* (100 mg/kg, equivalent to approximately 52 mg/kg periplocin).

**Results:**

The results showed that periplocin was absorbed rapidly in both groups (Tmax≤0.60 h). However, the extract matrix significantly altered the metabolic exposure. Compared with the periplocin group, the cardiac glycoside extract group demonstrated significantly higher levels of gomphogenin, with a marked increase in both C_max_ and AUC_(0-∞)_. Conversely, the systemic exposures AUC_(0-∞)_ of periplocymarin and 17*α*-asclepioside were significantly reduced. Furthermore, double peaks were observed in the concentration-time curves of gomphogenin and periplogenin, suggesting potential enterohepatic circulation.

**Discussion:**

These findings reveal that the complex matrix alters the bioavailability of active metabolites, providing a crucial kinetic basis for optimizing clinical dosage regimens and safety monitoring of *Cortex Periplocae*.

## 1 Introduction

The botanical drug *Cortex Periplocae* (Xiangjiapi), primarily distributed across the Northern, Northeastern, and Northwestern regions of China, is the dried root bark of *Periploca sepium* Bge. (Asclepiadaceae) (Chinese Pharmacopoeia Commission, 2025). Its principal active constituent is periplocin (C_36_H_56_O_13_), a major cardiac glycoside (Gao et al., 2019; Li et al., 2020) that has been widely documented to possess significant cardiotonic (Li et al., 2019; Zhao et al., 2023), antitumor, and anti-rheumatic effects (Xie et al., 2021; Lin et al., 2023; Wang et al., 2023; Zhang et al., 2020). Among them, particularly, the cardiotonic function of periplocin has attracted much attention (Li et al., 2016). It can selectively act on the heart to strengthen positive muscle strength by increasing myocardial contractility and slowing down the heart rate (Wang et al., 2021; Wang et al., 2010; Kahn and Schindler, 1962). Moreover, periplocin improves left ventricular structure and function and increases SERCA mRNA expression in rats with chronic heart failure (Hao et al., 2023).

The *in vivo* metabolites of natural products often play a pivotal role in their therapeutic efficacy. Consistent with this, periplocin has been reported to undergo biotransformation into periplocymarin and periplogenin in rat plasma following oral administration (He et al., 2015). Besides, periplocymarin has also been reported to have a strong cardiotonic effect and possesses higher exposure *in vivo* than the prototype drug periplocin (Yun et al., 2021a; Yun et al., 2020; Yun et al., 2021b; Yan et al., 2016). Meanwhile, periplogenin also plays a protective role in treating related cardiovascular problems and possesses anti-inflammatory activity (Panda and Kar, 2011; Gan et al., 2024; Ma et al., 2024). However, the complete metabolic profile of periplocin remains to be fully elucidated, and other potential active metabolites may also contribute to its pharmacological effects.

Pharmacokinetics describes the dynamic processes of absorption, distribution, metabolism and excretion which play a dominant role in elucidating pharmacological mechanisms (Xu et al., 2024; Zhao et al., 2024). While current research has focused mainly on periplocin or its primary metabolites, a critical gap remains since *Cortex Periplocae* is clinically administered as a complex extract rather than a pure compound (Wang et al., 2022). Given that existing components in botanical drug extracts are known to induce matrix effects comparative data between the complex extract and pure periplocin are still scarce. Consequently, the influence of the chemical matrix of *Cortex Periplocae* on the metabolic influence of periplocin requires further clarification.

To address this gap, this study aimed to investigate the influence of the multicomponent matrix of the cardiac glycoside extract on the *in vivo* absorption of periplocin. A sensitive and specific UPLC-TQ-S-MS/MS method was developed and validated to simultaneously quantify periplocin and its four key metabolites (periplocymarin, periplogenin, gomphogenin, and 17*α*-asclepioside) in rat plasma following the oral administration of either pure periplocin or cardiac glycoside extract from *Cortex Periplocae* at an equivalent dose. This research offers valuable insights into the potential component interactions within *Cortex Periplocae*, providing crucial kinetic data for clarifying its material basis and optimizing clinical dosage regimens.

## 2 Materials and methods

### 2.1 Reagents, chemicals, and materials

**FIGURE 1:**
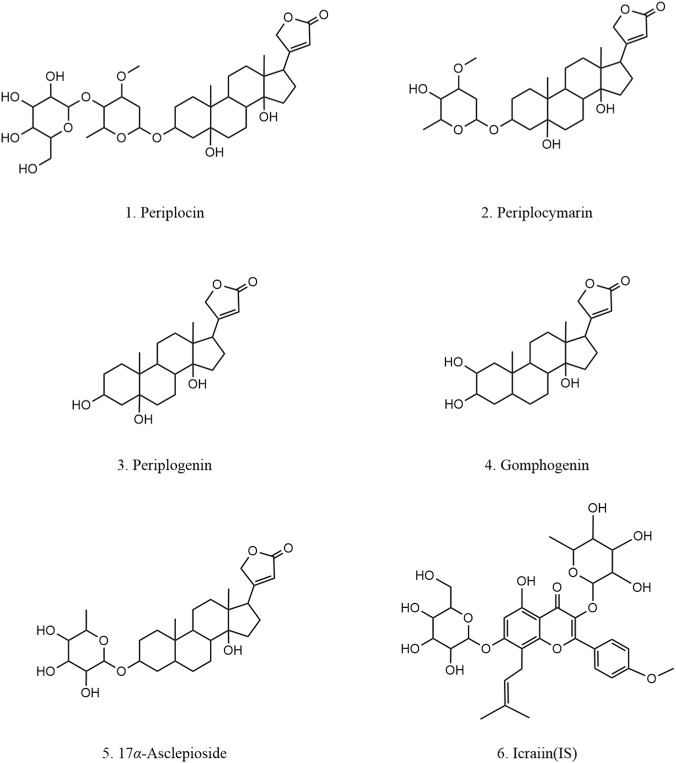
The chemical structures of five analytes and IS.

Methanol and acetonitrile of chromatographic grade were purchased from Fisher Chemical (Fairlawn, OSU, USA). Formic acid of chromatographic grade was purchased from Anaqua Chemicals Supply (Wilmington, DE, USA). Methanol of analytical grade was purchased from Concord Technology Co., Ltd., (Tianjin, China). Ultrapure water was produced by a Milli-Q water purification system (Millipore, Milford, MA, USA). Periplocymarin, periplocin and periplogenin were purchased from Chengdu Desite Biotechnology Ltd., (Chengdu, China). Gomphogenin and 17*α*-asclepioside were isolated and purified in our laboratory. Their chemical structures were confirmed by NMR and MS spectroscopies, and their purities were determined to be greater than 90% by HPLC analysis. The chemical structures of the 5 analytes and the IS were illustrated in Figure 1.

### 2.2 Targeted preparation of the cardiac glycoside extract

The botanical drug Cortex Periplocae (2.0 kg) was reflux-extracted three times with 6 volumes of 60% ethanol for 2 h each. The combined extracts were filtered, concentrated under reduced pressure, and lyophilized to yield the total extract. To enrich the cardiac glycosides, the total extract was dissolved in ultrapure water and subjected to AB-8 macroporous resin column chromatography, eluting sequentially with a gradient of ethanol-water (0%, 20%, 70%, and 100%, *v/v*). HPLC-DAD analysis indicated that the target compounds were primarily concentrated in the 70% ethanol fraction. This fraction was further purified using a Gilson GX-281 preparative liquid chromatography system equipped with an MCOLUMN-8C18A column (50 mm × 250 mm, 8 μm). The mobile phase consisted of 0.1% formic acid aqueous solution (A) and methanol (B) with a gradient elution at a flow rate of 50 mL/min. The target fraction, monitored at 230 nm, was collected, concentrated, and lyophilized to yield 2.5 g of the final cardiac glycoside extract.

### 2.3 Quality control and quantitative analysis

To ensure exact dose equivalence for the *in vivo* study, the extract underwent rigorous quantitative standardization using a Waters ACQUITY UPLC I-Class system coupled with a Xevo TQ-S tandem mass spectrometer. Chromatographic separation was achieved on an ACQUITY UPLC BEH C18 column (2.1 mm × 100 mm, 1.7 μm). Mass spectrometric detection was operated in positive ESI mode using multiple reaction monitoring (MRM). Calibration curves were established using pure periplocin (*r* > 0.999) and periplogenin (*r* > 0.999) reference standards. Comprehensive method validation, including precision, repeatability, stability, and recovery, met the accepted criteria. The quantitative analysis confirmed that the mass fractions of periplocin and periplogenin in the dried extract were exactly 51.94% (519.40 ± 7.99 mg/g) and 3.43% (34.29 ± 0.83 mg/g), respectively. This standardized extract was subsequently utilized for all pharmacokinetic experiments.

### 2.4 UPLC-xevo TQ-MS/MS condition

Waters UPLC-Xevo TQ-S-MS/MS (Santa Clar, CA, USA) was employed for analysis. The separation was performed by using a Waters ACQUITY UPLC®BEH C18 column (2.1 mm × 100 mm, 1.7 μm). The mobile phase containing 0.1% formic acid in water (A) and acetonitrile (B) was used to achieve the desired separation at a flow rate of 0.3 mL/min. The gradient program was as follows: 0–2 min, 5%–28% B; 2–7 min, 28%–43% B. The separation temperature was set at 35 °C. The samples were kept at 10 °C in the auto-sampler manager and the injection volume was 2 μL for analysis.

The mass spectrometer was operated in the positive ESI mode. Multiple reaction monitoring (MRM) was used for analysis. Nitrogen and argon were used as the nebulizer and the collision gas, respectively. The gas flows of desolvation and cone were 1,000 and 150 L/h. The desolvation temperature was set at 450 °C. The optimized MS parameters of the analytes were listed in Table 1.

**TABLE 1:** Mass spectrometry parameters of five analytes and IS.

Compound	Precursor ion (*m/z*)	Product ion (*m/z*)	Cone voltage (V)	Collision energy (eV)	Ionization mode
Periplocin	719.21	719.21	100	40	Positive
Periplocymarin	535.03	113.03	22	24	Positive
Periplogenin	391.01	355.16	22	8	Positive
Gomphogenin	391.08	355.16	23	10	Positive
17*α*-asclepioside	521.01	355.14	12	10	Positive
Icariin (IS)	677.08	531.17	60	12	Positive

### 2.5 Preparation of standard and quality control (QC) samples

Periplocin, periplocymarin, periplogenin, gomphogenin, 17*α*-asclepioside, and icariin (IS) were separately weighed and dissolved in methanol to a concentration of 1 mg/mL. Then, the working solutions were prepared by mixing an appropriate reserve solution of each analyte and diluting it with methanol to different concentrations. A mixture of 10 μL working solution of each analyte and 10 μL IS (100 ng/mL) was added to 100 μL blank plasma. Furthermore, the obtained mixture was extracted to obtain standard curve samples. The final concentrations of calibration standard solutions were in the ranges of 0.4-200 ng/mL (periplogenin), 3.2–1,600 ng/mL (periplocin, gomphogenin, 17*α*-asclepioside), and 8–4,000 ng/mL (periplocymarin). Three concentration levels of quality control (QC) samples were prepared in the same way. All the samples were stored at 4 °C before analysis.

### 2.6 Sample preparation

The rat plasma sample (100 μL) was successively spiked with 10 μL IS solution and 10 μL methanol. The mixture was extracted with 2 mL ethyl acetate, placed on a vortex mixer, shaken for 5 min, and centrifugated at 14,000 rpm for 10 min. The supernatant layer was transferred to a clean tube and evaporated to dryness under a nitrogen stream. The obtained residue was reconstituted with 100 μL of 50% methanol and centrifuged at 14,000 rpm for another 10 min. Finally, the 2 μL of the solution was injected into the UPLC-MS/MS system for analysis.

### 2.7 Method validation

The specificity was assessed by comparing chromatograms of blank plasma samples, blank plasma spiked with periplocin, periplocymarin, periplogenin, gomphogenin, 17*α*-asclepioside, and IS, as well as plasma samples obtained after oral administration of periplocin.

A calibration curve was created by plotting the peak area ratio of each analyte to the internal standard against its concentration, with a weighting coefficient of 1/X. The LOQ was the lowest concentration that could be measured with a signal-to-noise ratio (*S/N*) of 10.

Six sets of spiked QC samples at LOQ, low, medium, and high concentration levels were prepared and analyzed. The calibration curve constructed on the same test day was used for evaluation. The intra- and inter-day precisions were validated by calculating the relative standard deviation (RSD) value and the accuracy was expressed in terms of the relative error (RE) value.

The extraction recoveries were measured by comparing peak areas of analytes in extracted samples with those in post-extracted spiked samples. The matrix effects of rat plasma were obtained by determining the peak area ratios of the analytes in post-extracted spiked samples to the area of the peak in the standard solutions.

The stability was assessed by analyzing QC samples stored at room temperature for 4 h and in an auto-sampler for 12 h after preparation, −80 °C refrigerator for 7 days, and three freeze-thaw cycles.

All experimental data are expressed as the mean ± standard deviation (SD). The pharmacokinetic parameters were calculated using non-compartmental analysis with DAS 3.2.5 software. Statistical comparisons between the extract group and the periplocin group were conducted using GraphPad Prism version 10.0. For continuous pharmacokinetic variables, the independent-samples Student’s t-test was performed after confirming that the variables followed a normal distribution and exhibited homogeneity of variance. For variables that did not meet the assumptions of parametric tests, the Mann-Whitney U test was used. A *p*-value less than 0.05 was considered statistically significant.

### 2.8 Pharmacokinetic study

Male Sprague-Dawley rats (230 ± 10 g) were purchased from Beijing Huafukang Bio-Technology Co., Ltd. The rats were fasted for 12 h before the pharmacokinetics (PK) experiment and allowed free access to water during the experiment. Rats were randomly divided into two groups (n = 6 per group). To achieve the targeted doses at a constant administration volume of 10 mL/kg body weight, the test formulations were accurately prepared. Specifically, pure periplocin was suspended in 0.5% CMC-Na aqueous solution to yield a final concentration of 5 mg/mL. Similarly, the cardiac glycoside extract was suspended in the same vehicle to a concentration of 10 mg/mL. Consequently, oral gavage of these suspensions delivered exactly 50 mg/kg of pure periplocin and 100 mg/kg of the extract (equivalent to 51.94 mg/kg of periplocin), respectively. Blood samples (200 μL) were collected into heparinized centrifuge tubes from the fossa orbitalis at pre-dose, and 0.08, 0.25, 0.5, 0.75, 1, 2, 3, 4, 5, 6, 7, 8, 9, 10, 12, 24, 36, 48 and 60 h post-administration. After centrifugation at 6,000 rpm for 10 min, the liquid supernatant was transferred into a clean centrifuge tube and stored at −80 °C before analysis. To minimize pain and procedural distress, all blood collections were strictly performed under light isoflurane anesthesia. Furthermore, to mitigate the potential adverse effects of the cumulative blood volume drawn, the rats were supplemented with physiological saline solution after the first seven consecutive samplings (within 2 h) to maintain stable hemodynamics and physiological function. All animal studies were conducted under the guidance of Laboratory Animal Ethics Committee of Tianjin University of Traditional Chinese Medicine (TCM-LAEC2024032c1356).

## 3 Results

### 3.1 Method validation

**FIGURE 2:**
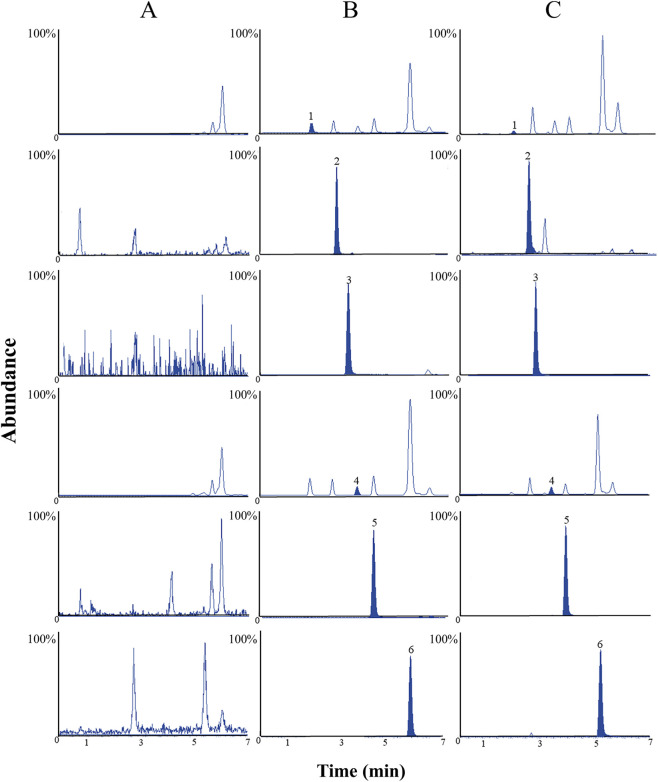
The MRM chromatograms of five analytes and IS **(A)** Blank plasma sample **(B)** blank plasma spiked with medium concentration QC **(C)** plasma sample after oral administration of periplocin. 1. Gomphogenin, 2. Periplocin, 3. Icariin (IS), 4. Periplogenin, 5.17*α*-asclepioside, 6. Periplocymarin.

Specificity has been studied by comparing chromatograms between blank plasma, blank plasma with medium QC, and plasma samples 5 h after oral administration of periplocin. Clearly, there was no significant interference at the same retention time of the analyte and IS. The results showed that these methods have good specificity (Figure 2).

As shown, calibration curves of all analytes had fine correlation coefficients (r) greater than 0.995 in a certain concentration range. The LOQs of periplocin, periplocymarin, periplogenin, gomphogenin and 17*α*-asclepioside were 2.0, 0.3, 0.1, 1.8 and 0.2 ng/mL, respectively (Table 2).

**TABLE 2:** The calibration curves, correlation coefficients (r), linear ranges, and LOQs of five analytes.

Compound	Calibration curve	Correlation coefficients (r)	Linear range (ng/mL)	LOQ (ng/mL)
Periplocin	Y = 0.00462245X + 0.0419384	0.997	3.2–1600	2.0
Periplocymarin	Y = 0.187357X − 1.92436	0.997	8.0–4000	0.3
Periplogenin	Y = 0.0922168X + 0.0831324	0.995	0.4–200	0.1
Gomphogenin	Y = 0.042735X − 0.106078	0.995	3.2–1600	1.8
17*α*-asclepioside	Y = 0.1322X − 0.145463	0.997	3.2–1600	0.2

The RSDs of intra-day and inter-day were less than 11.08%, the RE of intra-day was between −7.11% and 10.85%, while the RE of inter-day was between −8.44% and 9.80%. It indicated that the method had acceptable precision and accuracy (Supplementary Table S1).

The extraction recoveries of the analytes ranged from 80.09% to 110.87%, and RSD was less than 12.18%. This indicates that the extraction methods were consistent and efficient for extracting analytes in a wide concentration range from the plasma. The matrix effects of the analytes ranged from 80.43% to 117.83%, and RSDs were less than 12.90%. No endogenous component interference was found (Supplementary Table S2).

The stability results of storage at room temperature for 4 h, automatic sampler for 12 h, refrigerator at −80 °C for 7 days, and three freeze-thaw cycles were listed in Supplementary Table S3. All RSD values were less than 10.71%.

### 3.2 Pharmacokinetic study

**FIGURE 3:**
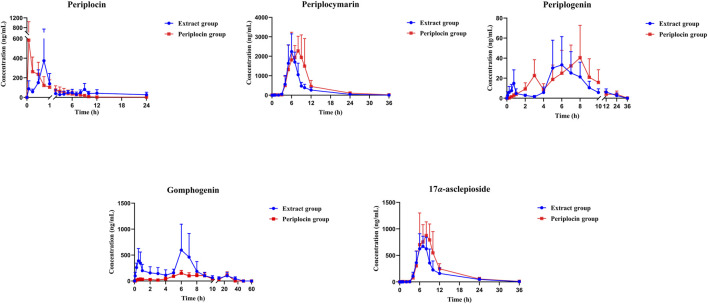
The concentration-time curves of analytes in rats plasma (*n = 6*, mean ± SD).

The pharmacokinetic parameters of periplocin and its four metabolites in the cardiac glycoside extract group and the pure periplocin group were calculated using the non-compartmental analysis model with DAS 3.0 software (Table 3; Figure 3). The results indicated that the parent drug, periplocin, was rapidly absorbed in both groups, with time to peak concentration (T_max_) values of 0.60 h in the cardiac glycoside extract group and 0.18 h in the pure periplocin group. In contrast, the biotransformation of periplocin into its metabolites was a relatively slow process. In both groups, the T_max_ values for periplocymarin, periplogenin, gomphogenin, and 17*α*-asclepioside were delayed to a range of approximately 6 to 8 h. Notably, the maximum concentration (C_max_) and systemic exposures AUC_(0-t)_ and ${\text{AUC}}_{\left(0-\infty \right)}$ of periplocymarin and 17*α*-asclepioside were substantially higher than those of the parent drug in both groups.

**TABLE 3:** The pharmacokinetic parameters of 5 analytes (*n* = 6).

Compound	T_max_ (h)		C_max_ (ng/mL)		T_1/2_ (h)		AUC_(0-tn)_ (h·ng/mL)		${\text{AUC}}_{\left(0-\infty \right)}$ (h·ng/mL)	
	A	B	A	B	A	B	A	B	A	B
C1	0.18 ± 0.17	0.60 ± 0.27^**^	657.56 ± 385.99	391.38 ± 357.61	2.81 ± 0.14	2.44 ± 0.27	703.02 ± 376.70	1346.12 ± 888.90	703.03 ± 376.70	1346.12 ± 888.90
C2	7.83 ± 1.60	6.33 ± 0.82	3,295.98 ± 400.70	2474.17 ± 572.39^*^	2.11 ± 0.14	2.32 ± 0.21	16,788.73 ± 3,166.31	10,787.97 ± 1587.89^**^	16,788.73 ± 3,166.31	10,787.97 ± 1587.89^**^
C3	6.83 ± 2.14	6.17 ± 1.17	51.73 ± 24.03	41.72 ± 23.05	2.71 ± 0.04	2.86 ± 0.16	282.32 ± 102.87	223.79 ± 117.73	282.32 ± 102.87	223.79 ± 117.73
C4	7.17 ± 1.72	6.33 ± 0.52	173.18 ± 27.31	740.58 ± 463.12^*^	2.08 ± 0.12	2.16 ± 0.27	2422.64 ± 805.89	4497.99 ± 2059.04^*^	2422.64 ± 805.89	4497.99 ± 2059.04^*^
C5	7.83 ± 1.60	6.83 ± 0.98	1233.97 ± 168.83	840.63 ± 190.08^**^	2.21 ± 0.10	2.30 ± 0.15	6,882.42 ± 614.24	4869.06 ± 1306.61^**^	6,882.42 ± 614.24	4869.06 ± 1306.61^**^

A. Periplocin group; B. Cardiac glycoside extract group; C1. Periplocin; C2. Periplocymarin; C3. Periplogenin; C4. Gomphogenin; C5. 17*α*-asclepioside.

Compared with the periplocin group.

* *P* < 0.05.

** *P* < 0.01.

However, comparative analysis revealed that the complex matrix significantly altered the quantitative exposure of these metabolites. Despite being the dominant components, the systemic exposures of periplocymarin and 17*α*-asclepioside were significantly suppressed in the cardiac glycoside extract group compared to the pure periplocin group, as evidenced by marked reductions in their C_max_ and AUC values (*P* < 0.01). In sharp contrast, gomphogenin exhibited the opposite trend, with its C_max_ and systemic exposure significantly higher in the cardiac glycoside extract group (*P* < 0.05). In addition to the changes in exposure levels, the plasma concentration-time curves of gomphogenin and periplogenin displayed distinct double peaks.

## 4 Discussion

The primary objective of this study was to elucidate the impact of the *Cortex Periplocae* chemical matrix on the *in vivo* disposition of its major bioactive component, periplocin. Our comparative pharmacokinetic analysis revealed that while the absorption of the parent drug was rapid and comparable between the pure periplocin and the cardiac glycoside extract groups, the systemic exposure of its metabolites exhibited significant divergence.

This indicates that the coexisting components in the extract do not significantly interfere with the initial absorption of periplocin but profoundly modulate its subsequent metabolic biotransformation and elimination processes. In both groups, T_max_ of metabolites (6–8 h) was significantly delayed compared to the parent drug (0.18–0.60 h). This temporal lag suggests that periplocin acts as a specific prodrug that undergoes extensive and sequential hydrolysis *in vivo* to form secondary glycosides and aglycones. The finding that periplocymarin and 17*α*-asclepioside exhibited higher systemic exposures than the parent drug aligns with previous reports suggesting that these metabolites may be the material basis for the sustained therapeutic effects of *Cortex Periplocae* (Li et al., 2019; Wang et al., 2022). Therefore, monitoring these major active metabolites is as crucial as monitoring the parent drug for safety and efficacy assessments. The most striking finding of this study is the bidirectional regulatory effect of the extract matrix on the metabolic profile of periplocin. Rather than simple competitive inhibition or the presence of additional precursors, our results point toward a more dynamic accelerated sequential turnover mechanism. Within the sequential biotransformation framework of periplocin, the systemic exposure of intermediate glycosides is dictated by the dynamic balance between their formation from the parent drug and their subsequent hydrolysis into the terminal aglycone (Wu et al., 2017). Our data reveals that the cardiac glycoside extract significantly suppressed the systemic exposure of intermediate glycosides (periplocymarin and 17*α*-asclepioside) while concurrently enhancing the terminal aglycone. This reciprocal alteration suggests that co-existing components in the *Cortex Periplocae* extract may selectively activate or induce the specific hydrolytic enzymes such as glycosidases, responsible for the conversion of these intermediates (Gerisch et al., 2018). Consequently, the intermediate glycosides are rapidly consumed and further hydrolyzed into the aglycone as soon as they are formed. This funneling of the metabolic flux effectively reshapes the intracellular metabolic equilibrium, leading to a diminished intermediate pool and a significantly expanded terminal aglycone pool. Such matrix-mediated modulation of metabolic rates underscores the sophisticated botanical drug interactions that define the holistic pharmacokinetic behavior of Traditional Chinese Medicine. In addition, the distinct double peaks observed in the concentration-time profiles of gomphogenin and periplogenin suggest the involvement of enterohepatic circulation (Watari et al., 1984). As lipophilic aglycones or secondary glycosides, these metabolites may be excreted into the bile and subsequently reabsorbed in the intestine. This recycling process prolongs their residence time in the body, which could contribute to the extended duration of action but also poses a potential risk of accumulation and toxicity (Chen et al., 2021; Zhou et al., 2019). It is important to note that this interpretation currently remains a hypothesis. A limitation of the present study is the absence of direct validation to confirm this recycling process. Consequently, other potential mechanisms, including segmented absorption across different regions of the gastrointestinal tract, cannot be definitively excluded. These pharmacokinetic phenomena warrant further investigation in future studies using targeted experimental models to fully elucidate the disposition and potential accumulation risks of these active metabolites. In addition, the current investigation was conducted following a single oral administration in healthy animals. Recognizing that *Cortex Periplocae* is clinically utilized for chronic conditions such as heart failure, future studies must evaluate the steady-state pharmacokinetics of these active metabolites following multiple-dose regimens in pathological models, thereby bridging the gap between preclinical kinetics and clinical safety.

Current clinical quality control of *Cortex Periplocae* largely focuses on the content of periplocin. However, our study demonstrates that the *in vivo* behavior of periplocin in the extract form differs significantly from its pure form due to matrix effects. The significant alteration in metabolite exposures implies that efficacy and toxicity profiles may vary between the periplocin and the extract. Therefore, pharmacokinetic markers for clinical monitoring should not be limited to periplocin alone but should include key metabolites like periplocymarin, 17*α*-asclepioside and gomphogenin to ensure a more comprehensive safety evaluation.

## 5 Conclusion

In conclusion, this study successfully developed and validated a sensitive UPLC-TQ-S-MS/MS method to systematically compare the pharmacokinetic profiles of periplocin and its four key metabolites following the oral administration of pure periplocin and the cardiac glycoside extract. The comparative results demonstrated that the multi-component matrix of the extract significantly modulated the *in vivo* disposition of the active ingredients. While the parent drug periplocin was rapidly absorbed in both groups, the extract matrix exerted a bidirectional regulatory effect on its metabolites: it significantly suppressed the systemic exposures of periplocymarin and 17*α*-asclepioside, whereas it markedly enhanced the exposure of gomphogenin. Additionally, the observation of double peaks in the concentration-time curves provided pharmacokinetic evidence for potential enterohepatic circulation or segmented absorption. These findings shed light on the complex internal botanical drug interactions within *Cortex Periplocae*, providing valuable kinetic data for clarifying its material basis and optimizing clinical dosage regimens to ensure safety and efficacy.

## Data Availability

The original contributions presented in the study are included in the article/Supplementary Material, further inquiries can be directed to the corresponding author.
