# Supplementary material for: Comparative pharmacokinetics of periplocin and its four key metabolites in rats after oral administration of periplocin and cardiac glycoside extract from Cortex Periplocae
Source: Front Pharmacol. 2026 May 14;17:1844009. doi: 10.3389/fphar.2026.1844009 (PMC13216200; doi:10.3389/fphar.2026.1844009)
Supplement: Supplementary file 1 [file DataSheet1.docx]

**Table S1** The intra- and inter-precision and accuracy of five analytes (*n = 6*).

| Compound | Concentration (ng/mL) | Intra-day | | Inter-day | |
| --- | --- | --- | --- | --- | --- |
|  |  | RE (%) | RSD (%) | RE (%) | RSD (%) |
| periplocin | 2 | 7.21 | 8.18 | 9.25 | 3.54 |
|  | 8 | 0.12 | 7.87 | -1.00 | 4.55 |
|  | 80 | 0.31 | 6.28 | -3.65 | 6.89 |
|  | 1280 | -2.11 | 2.21 | -4.66 | 8.62 |
| periplocymarin | 0.3 | -10.22 | 9.42 | 11.83 | 6.55 |
|  | 20 | 10.85 | 2.57 | 9.80 | 2.19 |
|  | 200 | 3.28 | 1.70 | 6.30 | 3.49 |
|  | 3200 | -7.11 | 8.11 | -8.44 | 4.07 |
| periplogenin | 0.1 | 5.41 | 8.56 | -3.37 | 9.53 |
|  | 1 | 2.00 | 4.90 | 4.00 | 6.73 |
|  | 10 | -0.20 | 5.01 | -0.90 | 1.61 |
|  | 160 | -2.39 | 4.35 | -4.08 | 11.08 |
| gomphogenin | 1.8 | 8.11 | 10.35 | 3.52 | 6.49 |
|  | 8 | 4.25 | 3.12 | 6.63 | 1.76 |
|  | 80 | -0.35 | 2.72 | 0.31 | 1.42 |
|  | 1280 | -0.38 | 6.35 | -6.52 | 7.71 |
| 17*α*-asclepioside | 0.2 | -6.35 | 9.33 | 8.65 | 10.35 |
|  | 8 | 2.50 | 3.90 | 7.00 | 1.52 |
|  | 80 | -2.95 | 6.49 | -1.54 | 2.63 |
|  | 1280 | -2.80 | 3.90 | -7.29 | 1.06 |

**Table S2** The extraction recovery and matrix effect of five analytes (*n = 6*).

| Compound | Concentration (ng/mL) | Extraction recovery (%) | RSD (%) | Matrix effect (%) | RSD (%) |
| --- | --- | --- | --- | --- | --- |
| periplocin | 8 | 102.94±3.13 | 3.04 | 117.83±10.53 | 8.94 |
|  | 80 | 87.09±4.43 | 5.08 | 111.40±10.87 | 9.76 |
|  | 1280 | 80.09±1.38 | 1.72 | 113.94±3.35 | 2.94 |
| periplocymarin | 20 | 92.97±4.54 | 4.88 | 105.58±6.40 | 6.06 |
|  | 200 | 92.65±3.07 | 3.31 | 102.86±5.68 | 5.52 |
|  | 3200 | 110.87±13.50 | 12.18 | 80.43±9.58 | 11.91 |
| periplogenin | 1 | 104.31±5.39 | 5.17 | 115.08±11.66 | 10.14 |
|  | 10 | 100.43±9.00 | 8.96 | 112.67±14.53 | 12.90 |
|  | 160 | 94.72±4.36 | 4.61 | 106.92±4.66 | 4.36 |
| gomphogenin | 8 | 104.83±2.08 | 1.98 | 89.09±1.44 | 2.56 |
|  | 80 | 97.49±2.85 | 2.70 | 100.99±3.72 | 3.68 |
|  | 1280 | 94.86±7.67 | 8.09 | 98.49±11.54 | 11.71 |
| 17*α*-asclepioside | 8 | 91.15±1.06 | 1.17 | 101.64±7.45 | 7.33 |
|  | 80 | 89.21±2.02 | 2.26 | 96.26±2.43 | 2.53 |
|  | 1280 | 80.48±5.30 | 6.58 | 93.62±4.44 | 4.75 |

**Table S3** The stabilities of five analytes (*n = 6*).

| Compound | Concentration (ng/mL) | Room temperature for 4 h | | Autosampler for 12 h | | Three freeze-thaw cycles | | −80°C for 7 days | |
| --- | --- | --- | --- | --- | --- | --- | --- | --- | --- |
|  |  | Measured (ng/mL) | RSD  (%) | Measured (ng/mL) | RSD (%) | Measured  (ng/mL) | RSD  (%) | Measured  (ng/mL) | RSD  (%) |
| periplocin | 8 | 8.03±0.63 | 7.85 | 8.17±0.26 | 3.18 | 8.01±0.39 | 4.87 | 7.79±0.32 | 4.11 |
|  | 80 | 71.47±3.12 | 4.37 | 80.25±5.04 | 6.28 | 81.53±3.85 | 4.72 | 82.36±3.62 | 4.40 |
|  | 1280 | 1266.7±61.60 | 4.86 | 1253.00±27.70 | 2.21 | 1147.90±65.10 | 5.67 | 1255.20±74.40 | 5.92 |
| periplocymarin | 20 | 25.59±0.86 | 4.16 | 21.78±0.60 | 2.75 | 22.91±0.57 | 2.49 | 21.63±0.55 | 2.54 |
|  | 200 | 233.28±7.55 | 3.92 | 214.33±8.08 | 3.77 | 229.60±17.40 | 7.57 | 202.09±2.27 | 1.12 |
|  | 3200 | 2997.00±177.20 | 5.91 | 2970.20±260.70 | 8.78 | 2897.40±91.10 | 3.14 | 3155.90±92.20 | 2.92 |
| periplogenin | 1 | 1.15±0.04 | 3.48 | 0.98±0.04 | 4.08 | 1.03±0.10 | 9.71 | 1.10±0.04 | 3.64 |
|  | 10 | 9.62±0.21 | 2.18 | 9.53±0.86 | 9.02 | 9.41±0.32 | 3.40 | 10.72±0.56 | 5.22 |
|  | 160 | 163.71±8.62 | 5.27 | 164.4±4.06 | 2.47 | 154.6±2.55 | 1.65 | 158.36±3.78 | 2.39 |
| gomphogenin | 8 | 8.29±0.31 | 3.74 | 8.44±0.35 | 4.15 | 8.14±0.16 | 1.97 | 7.92±0.39 | 4.92 |
|  | 80 | 75.76±2.21 | 2.92 | 81.86±5.25 | 6.41 | 74.41±1.87 | 2.51 | 78.62±2.30 | 2.93 |
|  | 1280 | 1303.50±59.40 | 4.56 | 1204.60±128.90 | 10.71 | 1267.70±70.90 | 5.59 | 1264.20±70.50 | 5.58 |
| 17*α*-asclepioside | 8 | 8.85±0.35 | 7.85 | 8.84±0.21 | 3.18 | 7.27±0.21 | 4.87 | 8.13±0.15 | 4.11 |
|  | 80 | 85.98±2.78 | 4.37 | 77.39±4.61 | 6.28 | 78.10±4.33 | 4.72 | 73.98±3.48 | 4.40 |
|  | 1280 | 1160.30±67.80 | 4.86 | 1186.40±51.10 | 2.21 | 1050.60±26.10 | 5.67 | 1214.30±59.20 | 5.92 |


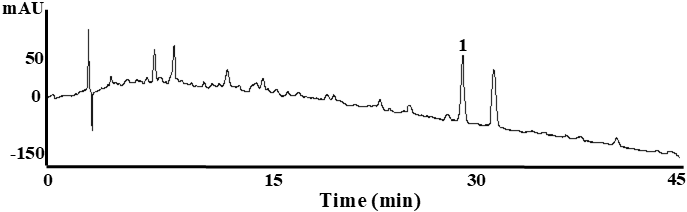


**Figure S1** Chromatograms of 70% ethanol fraction at 230 nm wavelengths


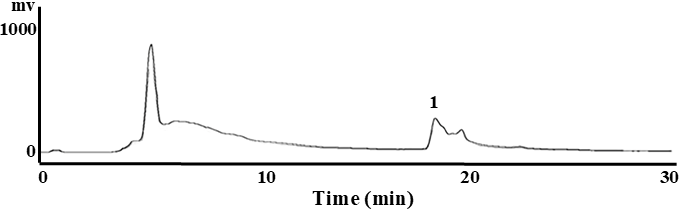


**Figure S2** Prepared liquid chromatogram of fraction


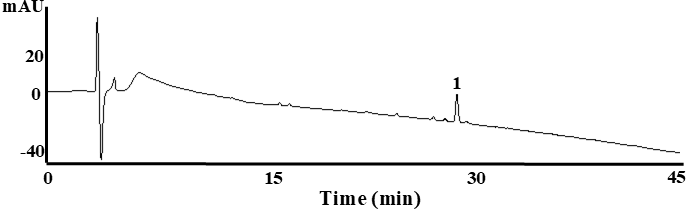


**Figure S3** Chromatograms of fraction at 230 nm wavelengths

Note: 1. periplocin


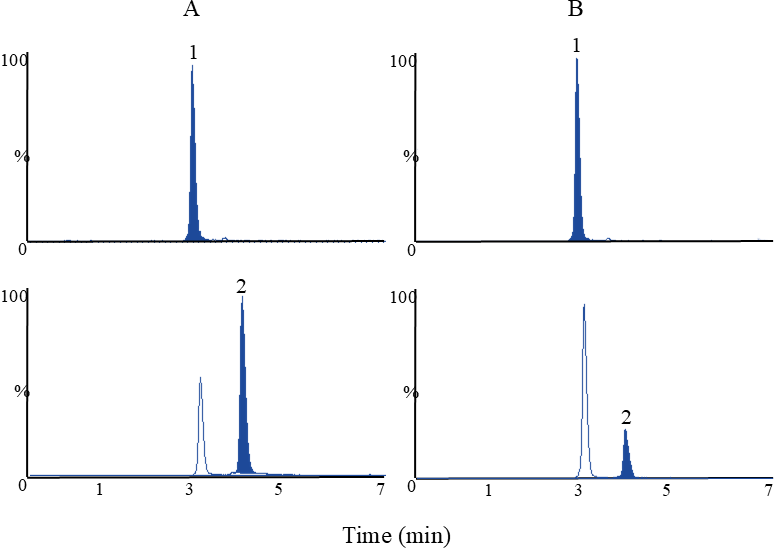


**Figure S4** chromatograms of periplocin and periplogenin

Note: A. Mixed reference sample diagram; B. Sample image; 1. periplocin; 2. periplogenin
